# The Authority

**Published:** 1876-04

**Authors:** Ben. H. Pratt


### THE BISTOURY.

Vol. XII. ELMIRA, N. Y., APRIL, 1876. No. 1.

### Contributions.

### THE "AUTHORITY."

BEN. H. PRATT.

In 1845, Astonville, though a very unpretending village in Lycoming County, in the state of Pennsylvania, possessed, however, a medical Authority of no mean order. Perhaps Astonville was not an exception to villages of its size and seclusion in this respect. From after experience in this particular we are inclined to think it was not. This Authority was always Authority—resident Authority; for it was many miles to the regular doctor's, and oftentimes emergencies arose which required immediate advice and action. The advice of this personage was not to be sneezed at, and there were few in the village who had the temerity to gainsay any of her diagnoses, or dispute the potency of any of her remedies. She had never, it is true, passed through any of the formulæ which are required to enable one to append M. D. to his name, and her stock of conventional medical lore would not have inspired a modern student of medicine with any profound adoration of her attainments; but she had grown up with the village, and from a period immemorial, had been consulted, at one time and another, by the wisest of Astonvillians, and not without success—so said her consulters, at all events. She had been present at every birth, ailment, accident and death that had occurred in the village. She took especial pains to assert this upon the very few occasions at which her abilities were questioned. The doctor who rode through the place once or twice a week, and who was always hailed by somebody for advice or treatment, was not one to dispute Mrs. Stebbins' abilities. He was not a man of many words, and he never scolded about "that old woman's interference" with the sick. Some were unkind enough to say that he was only too glad to have her around, as she helped business. This "helping business" is a significant term when applied to a doctor's practice. At all events, he never seemed to ignore her as many physicians do ignore such people, and never directly countermanded her draughts, applications or verbal prescriptions. "They can do no harm," was his usual response to inquiries as to the effect of Mother Stebbins' remedies, and upon several occasions he was known to say "best thing you could have done—certainly the very best." She sometimes was a trifle autocratic in her diagnosis, and although the old doctor never said anything when he knew she had made a mistake, yet they who knew him best could once and a while detect a queerish twinkle in his eye, or a peculiar drawing down of the corners of his mouth, which indicated that he knew a little more about some diseases than the Authority did. When he caught her in a very pointed error of judgment, he didn't puff himself up by berating her, but had a peculiar way of intimating these deficiencies without appearing to do so, and, which was a greater point with him, without offending his contemporary. One case in particular completely threw Mother S. off the track, and as it was one of the few sensational events of the village, impressed itself upon a certain boyish mind very forcibly, particularly as the boy was the cause of the sensation, and continued the subject of village talk for months after the occurrence of the event which we purpose briefly to narrate.

The son of the principal personage of the village, a boy about eight years of age, took his infant sister out one afternoon, in the baby-carriage. The day was one of Spring's very choicest, and the lad went up the main road leading north from the village. They had been gone about an hour, when the cries of the infant attracted the attention of somebody, who went in thedirection of the cries to discover the cause. About a quarter of a mile up the road the boy was found lying insensible, pale as a corpse, and huge beads of cold sweat oozing from every pore. It had evidently been some time since he had ceased to attend to the infant's cries. Beside him was a heap of a dark colored, almost black semi-liquid substance, evidently the result of copious vomiting. No efforts on the part of those who were summoned to the spot could bring about any return to consciousness. Limp, and cold, and clammy with perspiration, the boy lay, only his scarcely perceptible breathing and very feeble pulse indicating the presence of life. Hurriedly they bore him home, and "the Authority" was called in. She had no sooner arrived than the boy struggled once or twice convulsively, rolled over and threw from his stomach another mass of the same dark, viscous fluid, which had been so suspiciously noted at the place by the roadside, where the boy was found. All eyes were turned upon Mother Stebbins' face, and all who saw her knew that she was horror struck. She shrank from the child aghast—waved all from the couch on which he lay—hoarsely whispered, but loud enough for all to hear, "yellow fever. Don't you see that black vomit? Leave this house every one of you!" Even the mother of the boy was, for a moment, paralyzed with horror, but a mother's instinct overcame all save her love, and she caught her boy to her breast and screamed, "will no one go for the doctor?" Of this, no one had before thought, owing to the excitement and consternation to which Mother Stebbins' diagnosis had given rise. Then at once a dozen started off in hot haste for the doctor's home, ten miles away. But the medico was met five miles from the village, on his way to administer to the surgical needs of a millwright whose leg had that day been taken off by the mill saw. On the way the doctor was informed of the circumstances in the case, and though he wondered much at the recital, exhibited no especial surprise at the announcement Mother Stebbins had made. Arriving at the house he found it deserted by all save the boy's mother and "the Authority," while terror stricken knots of men and women had gathered at every available point, standing within sight of the window of the room wherein was confined one whose death from yellow fever all momentarily expected to hear announced. The doctor entered—felt of the child's pulse—noted the exceeding pallor of his face, and the claminess of his skin—then inquired after the matter ejected from the stomach. But this had been buried by the hands of "the Authority" herself, who feared contagion. Upon learning this fact, the doctor seized his hat and hastened on foot up the road to the spot where the boy was first found. He did not return so hastily as he went, and the villagers who were carefully watching his movements declared that his face did not betoken that extent of horror which the occasion seemed to call for. He did not smile at all, as he passed silently by the neighbors, but his features were far from serious in their make-up. He passed again that into the forsaken house—informed the mother she need not feel alarmed for the life of her boy—said that this disease was very seldom fatal—gave "the Authority" a look, and only said, "I can do nothing for the boy, and must hurry on to perform an amputation up the creek—do your best Mother Stebbins." She implored him to stay. He was inexorable. She confessed her ignorance of the treatment necessary in yellow fever, and begged him to leave some medicine, at least, and not go off at a time when the whole village was in so fearful a state of consternation. But he heeded her not—hurried out and off—leaped into his saddle and would have been out of sight in a minute had not one of the more curious bystanders seized his horse by the bit, and demanded to know the worst. Thereupon the doctor called his interlocutor aside, and leaning from his saddle, said: "Brown, have you any plug about you? If so, be mighty careful hereafter where you leave it when not in use;" and starting up suddenly, gave his horse full rein and was soon galloping up the road, leaving his puzzled inquirer standing stock still, with blank amazement stamped upon his face. Of course, all the neighbors, both men and women, gathered about Brown, anxious to known what the doctor had said; and they, learning his reply from the lips of Brown, also stood wondering whether the doctor had gone crazy or was scared into such an incoherent expression as he had just made.

And all that night they two, the mother and the Authority sat with the patient, doing nothing but watch and whisper and think and fear, passing a terrible night, the one full of hope, the other wrapped in despair. But the morning came and the child awoke—awoke with a smile on his face as he saw and knew his mother—spoke calmly, though feebly, and inquired the cause of her evident alarm. He felt no pain and had no fever, and save a thorough exhaustion, seemed quite himself. When told that he had been very ill, and when all the circumstances of the affair had been narrated, his face assumed a half-pained, half-puzzled expression, but he said nothing that could lead to any explanation of his sudden indisposition.—He was soon better, and being up, evinced a ravenous appetite, which was gratified with the simplest of food, but without stint; and while the boy was bolting his bread and milk, the doctor called on his way home. What he whispered into the boy's ear the mother never knew, but she did know that there was ever afterward a complete understanding between her son and the doctor, and a never healed misunderstanding between her boy and the Authority. The latter never knew what ailed the youth, and the neighbors never told her; but the doctor and the villagers, about the stove at "the store," of a winter evening, had many a hearty laugh, as over and over the story had to be told—with the injunction of secrecy so far as "Mother Stebbins' was concerned, always imposed upon any new hearer—the story of how the boy had found a plug of tobacco in the road, and having seen the farmers chew it, resolved to eat some himself, and did, with the results above narrated, which results were simply the overthrow of Mother Stebbins' absoluteness as a medical authority—one who couldn't tell the juice of pig-tail tobacco from black vomit, nor a boy's first chewing experience from a case of yellow fever.

